# Selection and demographic history shape the molecular evolution of the gamete compatibility protein bindin in *Pisaster* sea stars

**DOI:** 10.1002/ece3.1042

**Published:** 2014-03-31

**Authors:** Iva Popovic, Peter B Marko, John P Wares, Michael W Hart

**Affiliations:** 1 Department of Biological Sciences, Simon Fraser UniversityBurnaby, British Columbia, Canada; 2 Department of Biology, University of Hawai'iMānoa, Hawaii; 3 Department of Genetics, University of GeorgiaAthens, Georgia

**Keywords:** Bindin, concerted evolution, gamete recognition, positive selection, sexual conflict

## Abstract

Reproductive compatibility proteins have been shown to evolve rapidly under positive selection leading to reproductive isolation, despite the potential homogenizing effects of gene flow. This process has been implicated in both primary divergence among conspecific populations and reinforcement during secondary contact; however, these two selective regimes can be difficult to discriminate from each other. Here, we describe the gene that encodes the gamete compatibility protein bindin for three sea star species in the genus *Pisaster*. First, we compare the full-length bindin-coding sequence among all three species and analyze the evolutionary relationships between the repetitive domains of the variable second bindin exon. The comparison suggests that concerted evolution of repetitive domains has an effect on bindin divergence among species and bindin variation within species. Second, we characterize population variation in the second bindin exon of two species: We show that positive selection acts on bindin variation in *Pisaster ochraceus* but not in *Pisaster brevispinus*, which is consistent with higher polyspermy risk in *P. ochraceus*. Third, we show that there is no significant genetic differentiation among populations and no apparent effect of sympatry with congeners that would suggest selection based on reinforcement. Fourth, we combine bindin and cytochrome *c* oxidase 1 data in isolation-with-migration models to estimate gene flow parameter values and explore the historical demographic context of our positive selection results. Our findings suggest that positive selection on bindin divergence among *P. ochraceus* alleles can be accounted for in part by relatively recent northward population expansions that may be coupled with the potential homogenizing effects of concerted evolution.

## Introduction

Genes involved in reproductive incompatibilities have been a central focus of emerging research on patterns of genomic evolution in sympatric divergence and speciation (Schluter 2001; Coyne and Orr 2004; Via 2009; Lessios 2011; Nosil and Schluter 2011; Servedio et al. 2011). Selection on genes that underlie mating specificity and mate recognition may play an important role in the evolution of nongeographic barriers to gene flow between populations diverging in key phenotypic or life history traits that involve interactions between individuals, such as mating behavior (Martin and Hosken 2004), host choice (Hawthorne and Via 2001; Messina and Jones 2011), and mate signaling (color and vision, Kronforst et al. 2006; biochemical, Smadja and Butlin 2008), or interactions between gametes such as cognate proteins involved in fertilization specificity (Clark et al. 2006; Vacquier and Swanson 2011; Sunday and Hart 2013). Genes encoding compatibility proteins have been shown to evolve rapidly in diverse groups of animals and plants (Swanson and Vacquier 2002; Clark et al. 2006; Turner and Hoekstra 2008), and the rapid divergence of interacting proteins involved in reproduction has also been implicated in the maintenance of species boundaries during hybridization in mammals (Paysuer and Nachman 2005) and insects (Maroja et al. 2009), and in reproductive incompatibilities between closely related species of marine invertebrates (e.g., Vacquier 1998; Hellberg and Vacquier 1999; Zigler et al. 2005; Clark et al. 2009).

Among externally fertilizing marine species, direct biological interactions between the sexes are restricted to a few proteins expressed on the surfaces of male and female gametes (Lessios 2011). Therefore, gamete recognition proteins are obvious potential targets of selection and may have a primary role in the evolution of prezygotic reproductive isolation in the marine environment where geological or other physical barriers to gene flow are often absent, transitory, or porous in nature (Palumbi 1994; Lessios 2007; Turner and Hoekstra 2008). In addition to maintaining barriers among existing species, male-expressed reproductive ligands, in particular the sperm acrosomal proteins bindin and lysin, show signatures of positive selection for high interspecific sequence polymorphism in many, but not all, genera (Metz and Palumbi 1996; Biermann 1998; Zigler et al. 2005; Springer and Crespi 2007; Moy et al. 2008; but see Metz et al. 1998; Yang et al. 2000; Yang and Swanson 2002; Zigler and Lessios 2003, 2004; Hart et al. 2012) and have been shown to be targets of positive selection for population-level differences within species of sea urchins (Geyer and Palumbi 2003), mussels (Riginos et al. 2006), and sea stars (Sunday and Hart 2013; Hart et al. 2014) that broadcast spawn their gametes into the plankton. Evidence of diversification in response to population-specific selective pressures suggests that proteins involved in gamete recognition and fertilization may be important candidates in the origins of genetic differentiation across the genome, although the key selective mechanisms by which gamete recognition proteins evolve in different taxa remain unclear.

Research among echinoid genera has made notable progress in characterizing the role of selection on population- and species-level variation in bindin (Lessios 2011; Vacquier and Swanson 2011; Vacquier 2012). Bindin lives a double life by mediating sperm–egg adhesion and serving as a sperm ligand for fusion to the egg membrane (Vacquier and Moy 1977; Metz et al. 1994; reviewed in Hirohashi et al. 2008). Comparative analyses of bindin suggest that positive selection for high relative rates of amino acid substitutions and variation in the number and length of repetitive elements among alleles has acted to strengthen fertilization specificity in a species-specific manner (Biermann 1998; Palumbi 1999; Zigler 2008; Levitan and Stapper 2009) and that bindin divergence may be an important mechanism for reproductive incompatibility among species (Swanson and Vacquier 2002; Zigler et al. 2005). However, a key unresolved question involves the origins of bindin differences and reproductive isolation between closely related congeneric species: Do they arise initially in an early part of the speciation process as differences among conspecific populations, or long after the initial stages of speciation and the evolution of genomic or developmental incompatibilities by reinforcement against hybridization in secondary contact among divergent species? Some sea urchin genera (*Tripnuestes*, *Lytechinus*, and *Arbacia*; Zigler and Lessios 2003, 2004; but see Lessios et al. 2012) consist of ecologically differentiated species in which speciation events seem to correspond to shifts in habitat use or other ecological traits (e.g., Tomaiuolo et al. 2007; Lessios et al. 2012). Those examples implicate ecological speciation (Schluter 2009) as the original source of population genomic divergence and of postzygotic reproductive isolation, with selection against maladaptive hybridization or mating costs in secondary contact as a possible source of divergence at bindin and other loci involved in prezygotic reproductive isolation (Dobzhansky 1940; Geyer and Palumbi 2003; Servedio and Noor 2003; Coyne and Orr 2004; McCartney and Lessios 2004; Lessios 2007; Pinho and Hey 2010). In contrast, other sea urchin studies seem to rule out a significant role for sympatry, secondary contact, and reinforcement against hybridization in the evolution of bindin divergence and reproductive isolation (Calderón et al. 2009, 2010; Geyer and Lessios 2009; Pujolar and Pogson 2011; Lessios et al. 2012), and in these cases, an alternative source of selection leading to prezygotic reproductive isolation is needed to account for bindin divergence and speciation (Palumbi 2009; Lessios 2011).

Among the selective mechanisms put forward to explain such cases of nonecological speciation (Schluter 2009) associated with positive selection on gamete recognition loci are sexual selection and intersexual conflict over the rate of fertilization and polyspermy (Gould and Stephano 2003; Levitan and Ferrell 2006; Palumbi 2009). Directional selection among males under conditions of sperm competition may favor adaptations in male reproductive proteins for more efficient swimming, binding to the egg extracellular coat, penetration of the egg extracellular layers, and fusion with the egg membrane (see Levitan 2004; Palumbi 2009). If high rates of sperm contact often lead to fatal polyspermy for eggs (and low reproductive success for females), then females may experience antagonistic selection to counter adaptations among males and sperm by altering their egg surface sperm receptors (Frank 2000; Palumbi 2009). One possible result of this kind of sexual conflict is a coevolutionary “arms race” among sperm and egg compatibility loci, which can drive the evolution of high allelic variation and diversification within populations by frequency-dependent selection (Palumbi 1999, 2009; Gavrilets and Waxman 2002; Haygood 2004; Gavrilets and Hayashi 2005; Levitan and Ferrell 2006; Levitan and Stapper 2009; Tomaiuolo and Levitan 2010).

Differences in specific mating system variables have been used to explain variation in the susceptibility to polyspermy and strength of positive selection among lineages of closely related sea urchins (*Strongylocentrotus*; reviewed in Levitan 2006, 2008; Pujolar and Pogson 2011). Theory suggests that high sperm densities and strong sperm competition should lead to high rates of polyspermy and strong conflict between the sexes over the rate of sperm–egg contact. In contrast, under conditions of low sperm density and limited sperm competition, sexual conflict should be weak and both sexes should experience selection favoring high compatibility between all sperm and all eggs (Levitan 1993, 2006). Therefore, the form and intensity of sexual conflict and the signatures of positive selection may vary among species that commonly experience high-density spawning, high local sperm concentrations, and strong sperm competition, in comparison with species in which sperm competition and sexual conflict are predicted to be relatively weak (Levitan 2006, 2008; Pujolar and Pogson 2011).

To investigate the selective processes that may shape patterns of divergence at compatibility loci within and between congeneric species, we describe bindin gene structure for all three species of the sea star genus *Pisaster: P. ochraceus*, *P. brevispinus*, and *Pisaster giganteus*, and analyze the evolutionary relationships between the repetitive domains of the second bindin exon. Focusing only on this variable bindin region, we then investigate the patterns of positive selection among specific codons and lineages of bindin alleles in multiple populations of two of these species and compare them to the patterns of divergence in the mitochondrial *cytochrome c oxidase 1 (COI)* gene to understand how patterns of bindin variation within species differ between *P. ochraceus* and *P. brevispinus. Pisaster ochraceus* and *P. brevispinus* provide an excellent testing ground for distinguishing between alternative selective processes acting on bindin polymorphism. The current overlapping geographic distributions of *P. ochraceus* and *P. brevispinus* include allopatric and sympatric populations in the northeastern Pacific, and these species are also expected to experience different forms and intensities of sexual selection and sexual conflict. We predict that the intensity and extent of positive selection is stronger in *P. ochraceus*, whose life history characteristics are more consistent with a selective regime of high sperm competition and polyspermic conditions. If selection against hybridization is a significant source of selection for bindin divergence, an expected outcome of bindin evolution in response to reinforcement would be a pattern of bindin differentiation between *P. ochraceus* populations that are sympatric and allopatric with *P. brevispinus* populations, or higher rates of bindin evolution when species occur in sympatry compared to allopatry (Coyne and Orr 2004; Lessios 2007). Finally, we combine bindin and COI data in isolation-with-migration models to estimate locus-specific rates of gene flow between *P. ochraceus* populations and consider how population demographic history has contributed to patterns of spatial bindin variation in this species.

## Methods

### Study system

*Pisaster* species are broadly sympatric along the western coast of North America. *Pisaster ochraceus* (Brant 1835) has the widest range and occurs in wave-exposed rocky intertidal habitats at middle and lower tidal heights from Prince William Sound, Alaska, to Cedros Island, Baja California, Mexico (Lambert 2000). Adult individuals are abundant and can be found in high-density aggregations during feeding and spawning in the spring and summer months (Paine 1974). *Pisaster brevispinus* (Stimpson 1857) is the largest species in this genus but has a shorter range from Sitka, Alaska, to Santa Barbara, California (Lambert 2000). Individuals are commonly found at low spawning densities and are generally widely dispersed in low intertidal and subtidal habitats on sandy-mud substrates (Farmanfarmaian et al. 1958; Smith 1961). Both species have separate sexes. *Pisaster ochraceus* and *P. brevispinus* females generally contain mature oocytes (*P. brevispinus* 160–170 *μ*m; *P. ochraceus* 150–160 *μ*m; Strathmann 1987) and spawn during the months of May to August and March to August, respectively (Strathmann 1987). Interspecific fertilization can be induced under laboratory conditions (M. Hart unpubl. obs.); however, hybridization has not been reported in nature. Fertilized eggs develop into bipinnaria larvae that swim and feed in the plankton for several weeks (Strathmann 1987). Although the larval duration for *P. brevispinus* has not been documented, the larvae of *P. ochraceus* can stay in the water column for 6–8 weeks (Strathmann 1987) and are expected to travel long distances (Harley et al. 2006). *Pisaster giganteus* inhabit low rocky intertidal habitats (Farmanfarmaian et al. 1958) and are generally absent north of Monterey Bay, California (Ocean Biogeographic Information System; http://iobis.org); however, the range and abundance of this species is relatively understudied. Because *P. giganteus* is relatively rare, its geographic distribution is poorly known, and the bindin of this species is particularly difficult to sequence due to the highly homogeneous repetitive domain structure of the second bindin exon, our population comparisons focus on *P. brevispinus* and *P. ochraceus*.

### Population sampling

**Figure 1:**
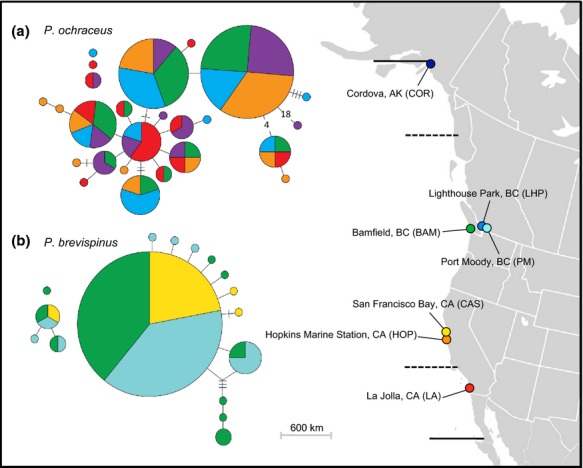
Collection sites for five *Pisaster ochraceus* and three *Pisaster brevispinus* populations sampled in this study are shown on the map on the right. Dashed lines represent the northern and southern range boundaries for *P. brevispinus*. Solid lines represent the range boundaries for *P. ochraceus*. Refer to Table 1 for exact localities and sample sizes. Bindin haplotype networks for (a) *P. ochraceus* and (b) *P. brevispinus* are shown on the left. Each circle represents a unique haplotype; the area of the circle is proportional to the frequency of the haplotype; colors show the proportion of each haplotype from different localities shown on the map. Each line between haplotypes represents one inferred mutational step, slashes indicate an additional mutation, and numbers within lines specify the number of additional mutational steps >3. Isolated haplotypes represent alleles that differed from the rest of the sample by insertion–deletion differences and could not be connected to the rest of the haplotype network by nucleotide substitutions only.

Samples were collected from a total of 36 *P. ochraceus* and 22 *P. brevispinus* individuals from eight localities from Alaska, British Columbia, and California (Table 1; Fig. 1). *Pisaster ochraceus* individuals were sampled from two populations from the northern (Cordova, AK) and southern (La Jolla, CA) extremes of its range where *P. brevispinus* has not been observed, and from three populations where it is sympatric with *P. brevispinus* (Bamfield, BC; Vancouver, BC; Hopkins Marine Station, CA)*. Pisaster brevispinus* is only found in sympatry with *P. ochraceus*; therefore, all populations that were sampled for this study coexist with *P. ochraceus*. We used *P. ochraceus* samples from Hopkins and La Jolla (Harley et al. 2006) and from Cordova (Marko et al. 2010) that were collected for previously published mtDNA surveys; *P. brevispinus* samples from California (San Francisco Bay) and a single *P. giganteus* sample used to obtain a full-length bindin allele sequence were obtained from the California Academy of Sciences, CA (Appendix S1, Table 1). All other samples were collected for this study from the field; tissues samples consisted of 10–20 tube feet preserved in 70–95% ethanol.

**Table 1:** Summary of collection sites and number of individuals and alleles sampled per location, per locus

Species	Location	Code	Coordinates	No. of individuals	Alleles sampled	

					Bindin	Cytochrome *c* oxidase 1
*Pisaster ochraceus*	Cordova, AK	COR	60.54°N 145.70°W	7	14	7
	Bamfield, BC	BAM	48.83°N 125.14°W	7	14	7
	Lighthouse Park, BC	LHP	49.33°N 123.26°W	7	14	7
	Hopkins Marine Station, CA	HOP	36.62°N 121.90°W	8	15	7
	La Jolla, CA	LA	32.84°N 117.28°W	7	13	7
*Pisaster brevispinus*	Bamfield, BC	BAM	48.83°N 125.14°W	8	16	8
	Port Moody, BC	PM	49.28°N 122.83°W	8	16	8
	San Francisco Bay, CA	CAS	37.72°N 122.28°W	6	8	4

### Genetic data

#### Species-level analyses: full-length bindin

**Figure 2:**
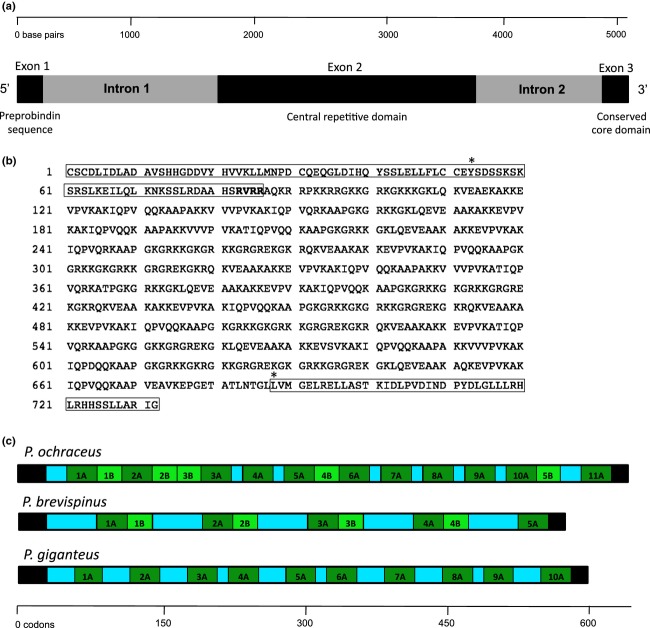
(a) *Pisaster ochraceus* bindin gene structure. (b) Inferred amino acid sequence for the full-length *P. ochraceus* bindin; the N-terminal preprobindin sequence and the C-terminal conserved core region are in boxes, and the furin-type cleavage site (RVRR) is in bold letters. Asterisks indicate splice sites for two introns. (c) Scaled diagram of repetitive domain structure variation in the second bindin exon for all three *Pisaster* species. Black regions represent nonrepetitive terminal sequences; collagen-like regions are shown in blue; *A* repeats are shown in dark green; *B* repeats are shown in light green and are absent in *Pisaster giganteus*.

For each individual sea star, we extracted genomic DNA from a single preserved tube foot using a proteinase K digestion with a 2× cetyltrimethyl ammonium bromide incubation (CTAB; Grosberg et al. 1996). To describe bindin gene structure, a single full-length bindin allele was amplified and sequenced for each *Pisaster* species. For *P. ochraceus* and *P. giganteus*, we used terminal primers CSCD and LAR (Table 2), which correspond to conserved amino acid motifs in the preprobindin and conserved core domain (respectively) of the sea star *Patiria miniata* (Patiño et al. 2009; also see Fig. 2a). An alternative preprobindin primer ADAV (Table 2) was designed to amplify bindin in *P. brevispinus*. Thermal cycling conditions for both sets of primers were 94° (1:00) for one cycle; 94° (0:15), 58° (0:30), 68° (5:00) for 33 cycles; and final elongation 70° (10:00). Amplicons were checked using 1% agarose gel electrophoresis and consisted of a single band of about 5 Kb (5027 bp in length for *P. ochraceus,* 4974 bp for *P. giganteus,* and 4859 bp for *P. brevispinus*). PCR amplicons were immediately cloned using a TOPO TA Cloning Kit (with PCR 4-TOPO vector; Invitrogen, Burlington, ON, Canada). Ten to 20 clones were screened for correct insert size, cleaned for sequencing using a DNA purification system (Wizard Plus SV Minipreps; Promega, Madison, WI), and sequenced using universal plasmid primers by a commercial sequencing service (http://www.operon.com). We confirmed the identity of those sequences based on blastx sequence similarities to *Patiria miniata* bindin (GenBank accession ACJ70121.1; Patiño et al. 2009). Custom forward and reverse primers were used to sequence along the remaining length of the gene (Table 2). Due to the highly homogeneous repetitive structure of the second bindin exon of *P. giganteus*, this gene region was not accessible with custom sequencing primers. Instead, we reamplified the second exon using the primers LRD and PGE, which correspond to the closest nonrepetitive amino acid motifs flanking the highly repetitive domain (Table 2). We used the same PCR cocktails, thermal cycling conditions, and cloning methods as described above and sequenced through the tandem repeats using universal plasmid primers. We characterized some general features of the bindin-coding sequence structure by searching for peptide cleavage sites in ProP (http://www.cbs.dtu.dk/services/ProP). We used the statistical analysis of protein sequences (SAPS; https://www.ebi.ac.uk/Tools/seqstats/saps/) package to estimate the molecular weight and amino acid composition of the predicted mature bindin molecule.

**Table 2:** Primer sequences for each locus and location in gene

Locus	Species	Primer name; direction	Primer oligonucleotides	Location in gene
Bindin	*Pisaster ochraceus*	CSCD; forward	5′-CCTGTTCGTGTGATCTGCTG-3′	Exon 1; preprobindin
		DET; forward	5′-GGATGAGACTTGTGGATGATTGCTT-3′	Intron 1
		AAV; forward	5′-AGGCTGCAGTCTGGTGACTT-3′	Intron 1
		DTL; forward	5′-GACACCCTCCCACTGTTTTCACTGT-3′	Intron 1
		LRD; forward	5′-GCAGTTTGAGAGATGCTGCTCATT-3′	Exon 2
		EVS; reverse	5′-TGCTTTAACTGAAACCTC-3′	Exon 2
		PGE; reverse	5′-TGTATTAAGGGTAGCCGTTTCACCAGG-3′	Exon 2
		TRQ; reverse	5′-AAGTGTGGGGAGCCTGTCTTGTG-3′	Intron 2
		LEL; reverse	5′-GTATAGGTTAGGGCTTGTGATAAGTAT-3′	Intron 2
		LAR; reverse	5′-TCACCAATGCGAGCTAGAAGACTGG-3′	Exon 3; core domain
	*Pisaster brevispinus*	ADAV; forward	5′-GCTGATGCAGTATCCCATCATGGTGAT-3′	Exon 1; preprobindin
		QLK; forward	5′-CCAGCTTAAGAACAAGAACAGTT-3′	Exon 2
mtDNA	*Pisaster ochraceus*	Poc-f-mt: forward^1^	5′-CTAATGATTGGCGCACCAGATA-3′	
		Poc-r-mt; reverse^1^	5′-GTAGTGAAAGTGGGCAACTACG-3′	

1 Harley et al. (2006).

#### Population-level analyses: second bindin exon

For the population-level analyses, we sequenced only the highly repetitive second exon in bindin where previous studies have documented highly significant population-level patterns of variation (*Patiria miniata*; Sunday and Hart 2013). In total, we cloned and sequenced 70 *P. ochraceus* and 40 *P. brevispinus* bindin alleles (Table 1). We designed two PCR primers, DTL and TRQ (Table 2), to amplify the second bindin exon for both *P. ochraceus* and *P. brevispinus* populations. Thermal cycling conditions used were 94° (1:00) for one cycle; 94° (0:15), 71° (0:30), 68° (2:00) for 26 cycles; and final elongation 70° (10:00). Amplicons consisted of a single band of 2154–2388 bp in length for *P. ochraceus* and 2144–2163 bp for *P. brevispinus* which correspond to the second bindin exon and about 50–200 bases pairs of 3′ and 5′ flanking introns. Six to 10 clones were screened for correct insert size and sequenced with forward and reverse universal plasmid primers. Alleles were inferred based on these sequences. Two or three copies of each inferred allele were sequenced with custom internal primers (Table 2) to minimize PCR and cloning errors, and unique nucleotide substitutions that differed from the consensus sequence of that allele were classified as sequence errors (see Sunday and Hart 2013). Additional alleles were sequenced if single-nucleotide differences or sequence ambiguities could not be resolved and true polymorphisms could not be confirmed by majority rule. Only one allele was sequenced and included in the data set for two *P. ochraceus* individuals in which nucleotide differences among the consensus bindin sequences indicated a second allele, but the full-length or true polymorphisms for that second allele could not be resolved. If sequences from all clones sequenced with plasmid primers had no polymorphisms, two to six clones were sequenced with internal primers to distinguish between potential alleles. If all sequenced clones represented the same allele, then this individual was considered a homozygote and two identical copies of this allele were included in the data set; PCR products from potential homozygotes were purified using Exo-SAP-IT PCR product cleanup kit (Affymetrix, Inc., Santa Clara, CA) and directly sequenced to confirm the absence of double nucleotide peaks. Chromatograms were proofread in 4Peaks v. 1.7.2 (A. Griekspoor and Tom Groothuis, mekentosj.com.). Two *P. brevispinus* individuals from San Francisco Bay could not be amplified, cloned, and sequenced with high quality; therefore, these samples were not included in the analysis.

We also amplified an 816-bp region of *COI* using primers from Harley et al. (2006). Although some *P. ochraceus* individuals from Hopkins and La Jolla were sequenced previously for COI by Harley et al. (2006), several of the sequences available from GenBank contained ambiguous nucleotide sites and the data were reported for only a 543-bp region of the gene. Therefore, we sequenced COI haplotypes for 35 *P. ochraceus* and 20 *P. brevispinus* individuals that were also sequenced for bindin in this study (Table 1; one Hopkins sample could not be amplified). The thermal cycling conditions used are described in Keever et al. (2009). All mtDNA sequences were checked on agarose gels, purified, and direct sequenced as described previously.

### Sequence alignment

Sequences of the second bindin exon were trimmed to the coding sequence only and visualized in Se-Al v2.0 (Rambaut 2002). Although bindin had strong sequence similarity within each species, length variation among some haplotypes complicated the alignment certainty among highly similar repetitive regions. We used a codon-based alignment algorithm in PRANK (Löytynoja and Goldman 2008; http://code.google.com/p/prank-msa/) to infer homology among codons, because the detection of positive selection can be sensitive to alignment error (Jordan and Goldman 2012). PRANK uses a codon model to align protein-coding DNA and has been shown to increase the accuracy of aligning truly homologous codons even in alignments of alleles with a high frequency of insertion–deletion differences (Jordan and Goldman 2012). We used PRANK to generate intraspecific alignments for *P. ochraceus* and *P. brevispinus* bindin alleles; we then compared them to alignments that included a single *P. giganteus* bindin allele as an out-group (sister species to *P. ochraceus;* Mah and Foltz 2011). All alignments generated by PRANK were manually inspected, and only minor adjustments were necessary. Because the second bindin exon is highly repetitive and varies in the number of repetitive domains among species (Fig. 2c), the orthology relationships between individual repeats are uncertain and we could not generate an interspecific haplotype alignment that included all three species with high confidence. Therefore, we limited our analyses to the intraspecific bindin alignments.

### Gene genealogies

We used the Model Selection tool implemented on the Datamonkey webserver (http://www.datamonkey.org; Delport et al. 2010) to infer the best-fit nucleotide substitution model for the bindin and COI haplotype data. The best model of molecular evolution (based on AIC comparisons) for each bindin alignment was determined to be F81 (Felsenstein 1981). For the COI alignments, the best-fit model of evolution was HKY85 (Hasegawa et al. 1985). The genetic algorithms for recombination selection (GARD) method for recombination detection in HyPhy (Kosakovsky Pond et al. 2011) was used to screen the bindin alignment, but GARD found no evidence of recombination in the second exon of bindin for either species.

Intraspecific bindin genealogies were constructed using maximum likelihood (ML) and Bayesian approaches. All duplicate haplotypes were removed, and the gene trees for both species were generated including and excluding a single bindin sequence from *P. giganteus* as a root. We used MEGA 5.0 (Tamura et al. 2011) to estimate an ML bindin gene tree for both species, under the most appropriate substitution model available (Jukes-Cantor). Gaps were treated as missing data (complete deletion), and branch support for the phylogeny was assessed by bootstrapping with 1000 replicates. Bayesian phylogenetic analyses were performed using MrBayes v. 3.1.2 (Ronquist and Huelsenbeck 2003). We applied an F81 substitution model and ran the two parallel Markov chain Monte Carlo (MCMC) searches, sampling every 1000 generations; after a burn-in of 200,000, a 50% majority rule consensus tree and posterior probability values of the nodes were estimated using the remaining trees from the posterior distribution. Run length and convergence of multiple chains were assessed using Tracer v.1.5 (Rambaut and Drummond 2007). Maximum likelihood and Bayesian methods generated almost identical topologies, with only minor differences in relationships between two terminal branches of the phylogeny; therefore, only the Bayesian gene trees are shown (Fig. 5). In addition, the intraspecific genealogies generated by both Bayesian and ML methods did not change when they were rooted with a *P. giganteus* sequence.

### Repetitive domain analyses

**Figure 3:**
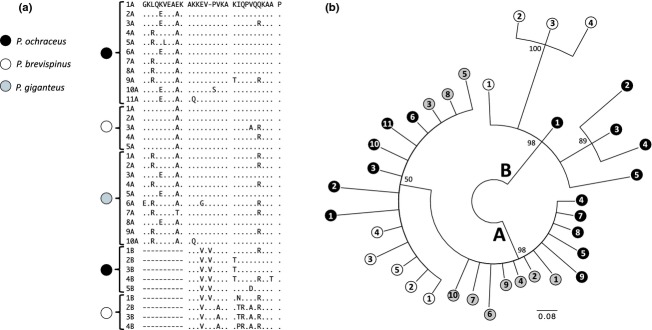
(a) Alignment of repeat domains in the second bindin exon for all three *Pisaster* species. (b) Consensus Bayesian genealogy of repetitive domains with Bayesian posterior probabilities of partition at branch nodes. “A” and “B” denote separate clades grouping repeat types *A* and *B*. Circles at branch tips are colored corresponding to species (see legend in panel a) and numbered corresponding to repeat number counted in the 5′ to 3′ direction.

We defined repetitive domains of the second bindin exon in RADAR (Heger and Holm 2000; Goujon et al. 2010) using the longest bindin allele from each species alignment and the single allele sequenced for *P. giganteus*. To investigate the evolutionary relationships between repetitive domains within and between species, we treated each repeat domain as a separate sequence and created an alignment of repeats within a single bindin allele and combined this alignment across all three species (Fig. 3a). We generated a phylogeny of repetitive domains using Bayesian reconstruction (Ronquist and Huelsenbeck 2003) and applied a HKY85 (Hasegawa et al. 1985) substitution model to the data (Fig. 3b). We looked for phylogenetic patterns among repeats consistent with an evolutionary mode of concerted evolution, in which the processes of gene conversion or misalignment and unequal crossover produce repeat paralogs from existing repetitive units within alleles (Smith 1976; Dover 1982; Elder and Turner 1995; Swanson and Vacquier 1998; McAllister and Werren 1999; Carmon et al. 2010). We looked for evidence for two key characteristics indicative of this process: (1) Repetitive domains are expected to have higher sequence similarity to each other within species, relative to repeats in corresponding regions of the coding sequence between species (Swanson and Vacquier 1998; Meeds et al. 2001); (2) unequal crossover events are expected to be suppressed closest to the unique sequences that are positioned at the ends of a repeating array (Stephan 1986); therefore, nucleotide substitutions that occur within terminal repeat domains are expected to be maintained and may lead to both higher sequence variation among terminal repeats relative to other internal repeat domains and greater sequence similarity between adjacent internal repetitive domains (McAllister and Werren 1999; also see Durfy and Willard 1989).

### Population genetic analyses

**Figure 4:**
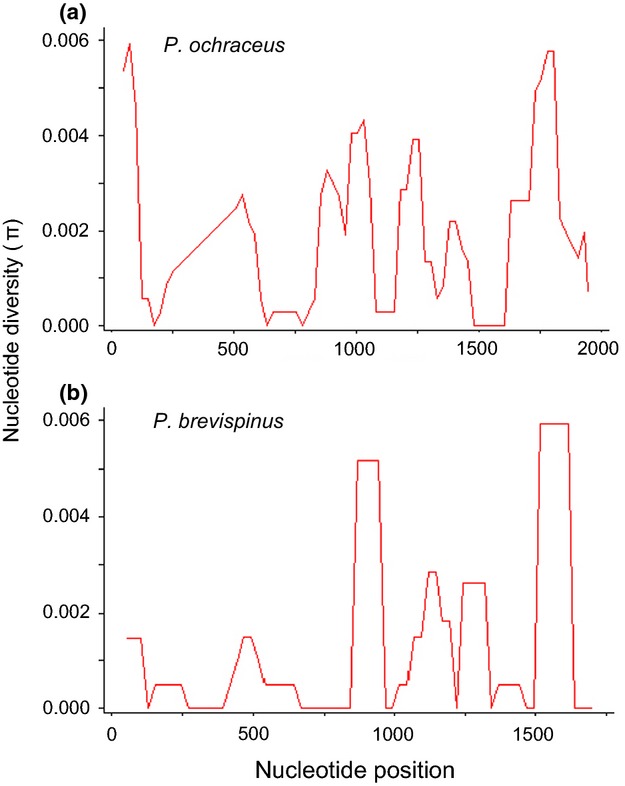
Variation in nucleotide diversity per base pair in sliding windows across the coding sequence for the second bindin exon for (a) *Pisaster ochraceus* and (b) *Pisaster brevispinus* populations (window length = 100; step size = 25).

To characterize population-level variation in the second bindin exon for *P. ochraceus* and *P. brevispinus*, we used DnaSP v. 4.10 (Rozas et al. 2003) to calculate nucleotide diversity from individual segregating sites (*π*) and haplotype diversity indices (*h*) including sites within alignment gaps. We also used the sliding window method to calculate and plot variation in nucleotide diversity per base pair across the bindin-coding sequence (window length = 100; step size = 25; Fig. 4). Allele frequencies, Tajima's *D* (Tajima 1989) values, and population differentiation in bindin and COI (*Φ*_ST_; Weir and Cockerham 1984) were calculated among all populations in Arlequin v. 3.11 (Excoffier et al. 2005). The most appropriate substitution models were applied to the data, and pairwise Φ_ST_ values were tested for significant departures from zero using 10,000 random permutations of the data. Bonferroni corrections were used to adjust probability values for multiple pairwise comparisons. TCS 1.21 (Clement et al. 2000) was used to construct and visualize parsimony networks of bindin and COI haplotypes using a 95% confidence of connection limit (Fig. 1).

### Tests of selection

We characterized selection acting on bindin-coding sequences using codon models that estimate the rate of nonsynonymous nucleotide substitutions per nonsynonymous site, d*N*, compared to the rate of synonymous substitutions per synonymous site, d*S*, among specific bindin lineages and codon sites, using the maximum likelihood method codeml in PAML 4.4 (Yang 2007). We used the unrooted intraspecific ML and Bayesian bindin phylogenies that consisted of only unique alleles. The branch-site method in codeml (Yang et al. 2005; Zhang et al. 2005) implements an ML model of codon evolution to compare *ω* = d*N*/d*S* among sets of foreground and background branches in the genealogy and identify individual codons, alleles, and lineages evolving under positive selection for high relative rates of amino acid substitutions with *ω* > 1 among lineages in the genealogy assigned to the foreground class (described in Yang et al. 2005; Zhang et al. 2005; Sunday and Hart 2013). The standard likelihood ratio test statistic (−2Δ*l*), twice the log-likelihood difference, was compared to the χ^2^ distribution (*P* = 0.05) to determine whether the selection model was a significantly improved likelihood fit to the sequence data, relative to the null model without a second set of codons under selection only on foreground branches. For all codeml analyses, we used pairwise deletion of sequence gaps to estimate d*N* and d*S* for sites within regions of the alignment that included insertion–deletion differences among some alleles.

We used the ML and Bayesian bindin genealogies for each species and performed a series of PAML analyses based on intraspecific population-level hypotheses to test a hypothesis of differential rates of positive selection among branches or among sites for each single population in the foreground, with all of the other populations in the background. For each population, we defined the foreground set of lineages as each of the terminal branches that lead to single alleles and any internal branches that lead to clades of alleles sampled from that population. Additional analyses to identify individual sites and branches under positive selection in bindin and COI were carried out using two methods implemented in HyPhy that employ ML-based analyses to fit different models of codon evolution and d*N*/d*S* rate variation to the sequence data. We used the mixed effects model of evolution (MEME; Murrell et al. 2012) method to detect the presence of diversifying selection on individual codon sites among some unspecified branches in the genealogies. The branch-site random effects likelihood (REL; Kosakovsky Pond et al. 2011) method was used to identify sets of lineages in the bindin gene trees that differ in the proportion of unspecified codons experiencing positive selection and a high average value of *ω*. These analyses were also carried out using the ML bindin topology and the most likely bindin gene tree generated from the Bayesian analysis to assess the effect of small differences among the tips of the topology on the inference of positive selection.

### Coalescent demographic models of gene flow

We used the isolation-with-migration coalescent population model in IMa (Nielsen and Wakeley 2001; Hey and Nielsen 2004, 2007; Kuhner 2009; Hart and Marko 2010 for review) to estimate effective rates of migration among conspecific *P. ochraceus* populations, for which we detected positive selection in our codon model analyses of bindin. Our analyses follow the multilocus approach of Sousa et al. (2013) and Hart et al. (2014) that use IMa-based methods to capture the effects of selection on rates of migration at loci evolving under positive selection (also see Bull et al. 2006). Such an approach allows fitting a neutral demographic model to estimate gene flow values at different loci across the genome, where relative reductions in effective migration rates reflect differences among populations in the relative fitness of immigrant alleles specific to those loci (compared to other loci not expected to be under selection; Sousa et al. 2013).

We performed a series of IMa analyses using different combinations of geographically adjacent *P. ochraceus* population pairs from north to south across the sampled geographic range. For each population pair, we combined the bindin haplotype data with sequence data for COI into a two-locus analysis (Data S2; also see Hart et al. 2014). We used an mtDNA mutation rate calibration of *μ* = 2.812 × 10^−6^ gene^−1^ year^−1^ based on a divergence rate of 4.56% per 3 million years estimated by Lessios et al. (2001) for the least-divergent sister species of *Diadema* and *Meoma* sea urchins separated by the Isthmus of Panama. This mutation rate has been used for coalescent analyses for other sea star species (McGovern et al. 2010; Puritz et al. 2012) and likely represents a conservative mutation rate estimate compared to other geminate Panamanian sea urchin species pairs that may have diverged from each other before the divergence of *Diadema* species (Knowlton and Weigt 1998; Marko 2002; Hickerson et al. 2006). The HKY finite sites mutation model was applied to the data (Hasegawa et al. 1985), and a generation time of 5 years was used as the average age of breeding in adults in these species of long-lived sea stars (Menge 1975; Strathmann 1987; also see Marko et al. 2010 and McGovern et al. 2010).

Several short runs were performed to identify the range of appropriate prior distributions for each parameter, and then priors were adjusted such that each posterior parameter distribution was contained within the lower and upper bounds of the prior distribution. We used strategies suggested by Jody Hey (http://genfaculty.rutgers.edu/hey/software#IMa; Using IMa documentation; Introduction to IM; and IMa documentation) and described elsewhere (e.g., McGovern et al. 2010; Marko and Hart 2011) to optimize the MCMC search such that the chains sufficiently “mix” or explore all of the possible highly probable genealogies and parameter values. We then used a sample of 50,000 gene trees saved from the last MCMCMC run in a series of likelihood ratio tests in “Load Trees Modes” or L-mode, in IMa to test hypotheses about differences among gene flow parameter values and ask whether the migration value estimates in both directions are significantly different from each other, and whether they are significantly different than the null hypothesis of zero gene flow (Hey and Nielsen 2007).

## Results

### Bindin gene structure

The full-length gene coding for bindin was highly similar in overall structure in all three *Pisaster* species and consisted of three exons and three protein domains: a cysteine-rich 5′ preprobindin region, a central repetitive domain, and the conserved 3′ functional core (Fig. 2a). The longest coding sequence from the conserved preprobindin amino acid motif CSCD to the core domain was 2196 bp (732 codons) in *P. ochraceus,* 2142 bp (714 codons) in *P. giganteus,* and 2034 bp (678 codons) in *P. brevispinus* (see Fig. 2b). The predicted amino acid sequence had high similarity to the preprobindin and core regions of *Patiria miniata* bindin (expectation value *E* = 2 × 10^−11^; Patiño et al. 2009). The 86-amino acid preprobindin sequence (Fig. 2b) is highly conserved among *Pisaster* species and contains just three nucleotide differences (between *P. brevispinus* and the other two species)*,* two of which confer nonsynonymous substitutions (codons 55 and 74). In addition to the CSCD amino acid motif, the preprobindin region contains two other cysteine residues (codons 31 and 50) that are shared with *P. miniata* (Patiño et al. 2009)*. Pisaster* preprobindin contains an intron ranging from 1677 to 1704 bp. Based on bindin cDNA from another forcipulate sea star, *Evasterias trochelii* (S. Patiño, unpubl. data), this intron is predicted to occur within a conserved tyrosine (codon 53) in the preprobindin sequence (Fig. 2b).

ProP identified a probable furin-type cleavage motif RVRR (codons 83–86) following the preprobindin region, identical to the furin-type cleavage site RVRR in the sand dollar *Encope stokesii* (Zigler and Lessios 2003) and similar to the RARR motif in *P. miniata* (Patiño et al. 2009) where the N-terminal preprobindin sequence is predicted to be cleaved from the mature molecule in the sperm acrosomal vesicle (Fig. 2b; Gao et al. 1986; Zigler and Lessios 2003; Patiño et al. 2009). The mature peptide downstream of the cleavage site is predicted to be 70 kDa, slightly smaller than the estimated size of the presumed bindin protein isolated from the acrosomal vesicle of *P. ochraceus* sperm (95 kDa; Christen 1985). The most common amino acids in mature bindin are lysine (26.3%), alanine (13.2%), and glycine (10.7%). *Pisaster* mature bindin contains no cysteine or tryptophan residues, similar to other echinoderm taxa for which the mature bindin protein has been sequenced (Zigler and Lessios 2003; Patiño et al. 2009).

Mature bindin gene structure is dominated by a central repetitive region (described below) that is located in the second exon between the preprobindin sequence and the core domain and varies among species in the number of repeating domains defined in RADAR. We found 13–16 repeats in *P. ochraceus*, 10 repeats in *P. giganteus,* and nine repeats in *P. brevispinus*. In spite of variation in the number of repetitive domains, the overall length of the second bindin exon and the repetitive nature of the gene structure were relatively conserved among species (Fig. 2c). Species also varied in the length of glycine- and lysine-rich collagen-like regions that separated the other parts of the repetitive domains (Fig. 2c). The collagen-like regions consisted of variable numbers of GKGRKK repeats that are similar to the much longer collagen-like domains in the bindin of *P. miniata*.

The predicted bindin core domain immediately downstream from a conserved leucine residue (also the predicted splice site for the second intron of 1027–1038 bp) had high sequence similarity to the invariant core region of *P. miniata,* with only a singleton amino acid difference (a serine–asparagine polymorphism) that is conserved in charge and polarity, and was similar to the central core region of several sea urchin species with the lowest expectation value of *E* = 4 × 10^−4^ when compared to *Pseudoboletia* sp. (AFB82032.1). The 43 amino acid residues of the core domain were very highly conserved among all three *Pisaster* species, with only one synonymous nucleotide substitution in *P. brevispinus*. The LGLLLRHLRHH amino acid motif, corresponding to part of the “B18” core domain of sea urchins, was identical among *Pisaster* species and identical to the same B18 regions of all sea urchin taxa (Zigler and Lessios 2003; Vacquier 2012) and other sea stars (Patiño et al. 2009) in which bindin has been sequenced (Vacquier and Swanson 2011). This domain has been shown to have a functional role in sperm–egg membrane fusion (Zigler 2008; Vacquier 2012) and has been predicted to be the binding substrate for the sea star egg bindin receptor OBi1 (Hart and Foster 2013; Hart et al. 2014).

### Repetitive domain analysis

RADAR identified all noncollagen repetitive domains as a single repeat type (see Fig. 3a). Repetitive domains of *P. ochraceus* and *P. brevispinus* (but not *P. giganteus*) included both long repeats (30 codons; dark green, labeled “*A*” in Fig. 2c) and short repeats (20–21 codons; light green, labeled “*B*” in Fig. 2c). Bayesian phylogenetic analyses showed that *A* and *B* repeat domains were grouped into two strongly supported clades (Fig. 3b). The overall similarity in gene structure among species, with multiple copies of distinct repetitive domain types, suggests that this repetitive structure is ancestral for the three *Pisaster* species and that repeat copies in the same location within the gene structure across species might be orthologs and more closely related to each other, than to paralogous repeat copies in other parts of the same allele. Instead, we found that the *A* repeats had overall high homogeneity among all repeat copies, with no strong pattern of higher or lower sequence similarity among repeats within species compared to between species. Low variation among *A* repeats (ranging from 91% to 100% sequence similarity) limited the resolution of phylogenetic relationships between repeat copies within clade *A* (Fig. 3b). Therefore, the distinction between *A* repeats that are orthologous and originated in a common ancestor and the repeats that originated independently within each species by unequal crossover or another molecular process could not be inferred. The genealogy of repetitive domains, however, showed that *B* repeats are more similar to each other within species than between species, with the exception of the first *B* repeat (1*B*) in *P. brevispinus,* which is more closely related to *B* repeats found in *P. ochraceus* (Fig. 3b). Among the five *B* repeats found in *P. ochraceus,* the three middle repeats (2*B*, 3*B*, and 4*B*) grouped into a single clade (Fig. 3b), consistent with the predicted effects of concerted evolution (Elder and Turner 1995; Swanson and Vacquier 1998; McAllister and Werren 1999; Meeds et al. 2001).

### Population genetic diversity

Bindin alleles differed by insertion–deletion mutations that produced variation in the length of the second exon ranging from 1728 to 1962 bp in *P. ochraceus,* including five individual heterozygotes with insertion–deletion differences between alleles involving part or all of one or more *A* or *B* repetitive domains or collagen-like sequence motifs (GKGRKK). We found fewer length differences in our smaller sample of *P. brevispinus* individuals and alleles. All *P. ochraceus* individuals were heterozygous for the second exon of bindin; the aligned coding sequence was 1980 bp (660 amino acids) with 35 variable nucleotide sites and 26 amino acid polymorphisms (Table 3). We found extensive allele sharing among populations; of 70 sequenced bindin alleles, there were only 25 haplotypes and only 13 gene copies were not shared among populations. The *P. brevispinus* bindin alleles ranged in length from 1719 to 1737 bp and varied only in the number of collagen-like sequence motifs (GKGRKK); the alignment was 1737 bp (579 amino acids) and was slightly less polymorphic with 17 variable sites and 14 amino acid polymorphisms (Table 3). Of 20 *P. brevispinus* individuals sequenced, four were homozygous. Among 40 sequenced alleles, there were 16 haplotypes. One common haplotype was found at a relatively high frequency (*n* = 18); this haplotype was inferred as ancestral by TCS and was present in all three sampled locations (Fig. 1b).

**Table 3:** Polymorphism statistics (including sites with gaps), number of variable nucleotide and amino acid sites, number of unique haplotypes, nucleotide diversity (*π*; at individual sites), haplotype diversity (*h*), and number of alleles with indel variation

Locus	Species	Location	Variable sites		Unique haplotypes	π	*h*	Alleles with indel variation
							
			*Nucleotide*	*amino acid*				
Bindin	*Pisaster ochraceus*	COR	16	13	10	0.00178	0.9451	2
		BAM	16	12	9	0.00183	0.9231	0
		LHP	20	13	9	0.00235	0.9231	1
		HOP	20	16	11	0.00249	0.9333	0
		LA	22	15	11	0.00199	0.9615	2
		All populations	35	26	25	0.00209	0.9346	5
	*Pisaster brevispinus*	BAM	11	10	9	0.00210	0.8167	3
		PM	11	8	8	0.00148	0.8000	3
		CAS	9	7	5	0.00131	0.7857	1
		All populations	17	14	16	0.00124	0.7897	7
mtDNA	*Pisaster ochraceus*	COR	10	0	4	0.00710	0.8571	0
		BAM	2	0	3	0.00093	0.5238	0
		LHP	1	0	2	0.00035	0.2857	0
		HOP	3	1	3	0.00175	0.7143	0
		LA	3	0	4	0.00146	0.8095	0
		All populations	13	1	7	0.00285	0.6689	0
	*Pisaster brevispinus*	BAM	7	1	4	0.00279	0.7500	0
		PM	10	0	5	0.00502	0.8571	0
		CAS	3	0	3	0.00306	0.8333	0
		All populations	14	1	7	0.00389	0.8316	0

Haplotype diversity (*h*) was high within all populations across the geographic range studied, but slightly higher in *P. ochraceus* populations (*h* = 0.9231–0.9615) than in *P. brevispinus* populations (*h =* 0.7857–0.8167). Nucleotide diversity was low and ranged from 0.00131 to 0.0.00249 among all populations (Table 3). Across all of the populations sampled, we observed the highest nucleotide diversity (∼0.006) contained within the C- and N-terminal repeat domains in *P. ochraceus* and the C-terminal repeat domain in *P. brevispinus* (Fig. 3c). In both species, Tajima's *D* values were generally negative (Appendix S1, Table 2), which may reflect an excess of rare alleles indicating population expansions or a signature of positive or purifying selection; however, these statistics were not significantly different from neutrality (*P* > 0.1) in all but two southern localities (Appendix S1, Table 2.). There were no fixed nucleotide or indel differences among populations. Pairwise population differentiation (*Φ*_ST_) was generally low (−0.05023 to 0.04830 in *P. ochraceus;* −0.006 to 0.07935 in *P. brevispinus*) and not significantly different from zero in any pairwise comparisons (Appendix S1, Table 3a), including *P. ochraceus* populations that are sympatric or allopatric with a congener.

Although the mtDNA sample sizes in this study were relatively small, we found sequence diversity among COI haplotypes in *P. ochraceus* similar to those in Harley et al. (2006) (see Table 3). Both species were characterized by high COI homogeneity (*Φ*_ST_ not significantly different from zero; Appendix S1, Table 3b).

### Selection analyses

**Figure 5:**
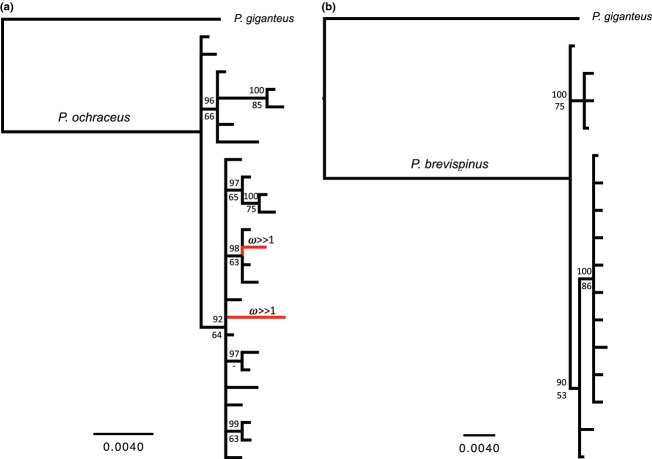
Consensus Bayesian genealogies of bindin alleles for (a) *Pisaster ochraceus* and (b) *Pisaster brevispinus* rooted with a single *Pisaster giganteus* bindin allele. The scale bar shows 0.4% sequence divergence. Numbers above branches represent Bayesian posterior probabilities of partition, and numbers below the branches denote bootstrap support of the maximum likelihood analysis from 1000 replicates. Red branches were inferred to be experiencing episodic diversifying selection with *ω* ≫ 1, using the branch-site random effects likelihood method implemented in HyPhy. Also refer to Tables 4 and 5 for additional tests of positive selection.

Codon models revealed a weak but statistically significant signal of positive selection (*ω* > 1) favoring a high relative rate of amino acid substitution differences among *P. ochraceus* bindin alleles. MEME identified two sites (codons 557 and 624) in both ML and Bayesian bindin gene trees to be under strong positive selection in the *P. ochraceus* alignment (Table 4). The branch-site REL analysis inferred two terminal branches in *P. ochraceus* to be under strong positive selection (*ω* ≫ 1, i.e., a very large but imprecisely estimated value); the first was a private allele found at Hopkins, and the second lineage was a highly derived allele (based on a haplotype network inferred in TCS with a 95% confidence limit) sampled only once in both La Jolla and Cordova (*ω* = 1256) (Table 4; Fig. 5); this particular allele contained an eight-amino acid deletion following a lysine–serine substitution at the positively selected site at codon 624. Branch-site models in PAML (Table 5) corroborated the tests of selection in MEME and estimated a high mean d*N*/d*S* ratio across a small proportion (0.5%) of codons with *ω* > 1, when alleles sampled from Cordova were in the foreground and all other populations were constrained as background lineages in the ML (*ω* = 197) and Bayesian (*ω* = 455) topologies. Both analyses identified one site (codon 624) with a posterior probability >95% for belonging in a separate rate class with *ω*2 ≫ 1. Tests for positive selection among *P. ochraceus* alleles sampled in Hopkins and La Jolla also yielded a significantly better fit to the data compared with the null model in both the ML and Bayesian topologies and estimated a high foreground rate of positive selection (*ω* ranging from 44.8 to 107) among a few codons. However, these analyses could not identify any individual sites with a high posterior probability of assignment to that second class of positively selected sites (Table 5).

**Table 4:** Summary of results from mixed effects model of evolution (MEME) and branch-site random effects likelihood (REL) tests of positive selection, implemented in HyPhy

Species; Analysis	Genealogy	Positively selected codons	Branches with *ω* > 1
		MEME (*P* > 0.1)	Branch-site REL (*P* > 0.5)
*Pisaster ochraceus*	Maximum likelihood (ML)	557V, 624K	2 lineages
	Bayesian	557V, 624K	2 lineages
*Pisaster brevispinus*	ML	0	0
	Bayesian	0	0

**Table 5:** Summary of branch-site models implemented in PAML. All codons inferred to be under selection correspond to sites within each separate species alignment; for these tests, we used a critical posterior probability value of *P* > 0.90

Species; Analysis	Genealogy	Branch-site hypothesis	−2Δln*L* (df = 1)	Parameter estimate (*ω*)	Positively selected sites (*ω* > 1; *P* > 0.90)
*Pisaster ochraceus*; Population-level	Maximum likelihood (ML)	Cordova	8.34^1^	*ω*2 = 197	624K (*P* = 0.948)
		Bamfield	0	*ω*2 = 1	–
		Lighthouse Park	0	*ω*2 = 1	–
		Hopkins	5.11^1^	*ω*2 = 44.8	–
		La Jolla	4.23^1^	*ω*2 = 77.4	–
	Bayesian	Cordova	9.99^1^	*ω*2 = 455	624K (*P* = 0.971)
		Bamfield	0	*ω*2 = 1	–
		Lighthouse Park	0	*ω*2 = 1	–
		Hopkins	5.03^1^	*ω*2 = 47.0	–
		La Jolla	5.12^1^	*ω*2 = 107	–
*Pisaster brevispinus*; Population-level	ML	Bamfield	2.18	*ω*2 = 188	–
		Port Moody	0	*ω*2 = 1	–
		SF Bay	1.24	*ω*2 = 1	–
	Bayesian	Bamfield	0	*ω*2 ≫ 1	–
		Port Moody	0	*ω*2 = 1	–
		SF Bay	0	*ω*2 = 1	–

1 Significant (df = 1).

The MEME and branch-site REL methods did not identify any specific sites or branches with *ω* > 1 among bindin lineages within *P. brevispinus* (Table 4). The series of branch-site codon models that compared signals of positive selection among *P. brevispinus* populations could not reject the null model in any likelihood ratio tests when each population was specified in the foreground (Table 5).

### Coalescent demographic analysis

Multilocus analyses among five geographically adjacent *P. ochraceus* population pairs (Table 6; Data S1) sampled in this study revealed directional migration parameters (*m*_1_, *m*_2_) for all population pairs that had the highest posterior probabilities (the modes in the posterior probability distributions) for the lowest bin in the posterior distribution, with the proportion of gene copies that are new migrants every generation ranging from 0.0000097 to 0.0000209 among all populations. However, because all probabilities for gene flow parameter estimates were relatively low, and low nonzero probabilities were estimated for a wide range of very high (and very low) gene flow parameter values, it was necessary to use relatively wide prior distributions to characterize the posterior probability distributions. Therefore, the posterior probability distributions for most migration parameter estimates had poorly defined upper and lower bounds (Appendix S1, Fig. S1). Likelihood ratio tests in L-mode yielded significantly poorer fits to all seven nested models that were fixed for the null gene flow parameter (*m* = 0) for all population pair analyses, with the exception of one population pair in which *m* estimates were not significantly different from *m* = 0 (immigration into Lighthouse park from Bamfield, *P. ochraceus*). The L-mode results, coupled with weakly defined lower bounds for migration parameters (including zero) and the absence of clearly defined posterior distributions with nonzero highest likelihood modes, suggest that gene flow is generally very low among populations on the spatial scale sampled in this study for both species.

**Table 6:** Summary of gene flow parameter estimates between *Pisaster ochraceus* populations inferred by IMa. Maximum likelihood estimates for the proportion of migrants into population 1 (*m*_1_) and population 2 (*m*_2_) per gene copy per 1000 generations, with confidence intervals (90% highest posterior densities, HPD) in parentheses

Species	Population pair	*m*_1_	*m*_2_
*Pisaster ochraceus*	COR—BAM	0.0118 (0.0118–20.36)	0.0177 (0.0177–29.48)
	COR—LHP	0.0139 (0.0139–23.36)	0.0139 (0.0139–23.86)
	BAM—LHP	0.0209 (0.0209–33.53)	0.0209 (0.0209–33.82)
	LHP—HOP	0.0209 (0.0209–34.74)	0.0209 (0.0209–35.12)
	HOP—LA	0.0097 (0.0097–16.18)	0.0097 (0.0097–16.00)

## Discussion

Genes encoding reproductive compatibility proteins have been shown to evolve rapidly under positive selection and have been implicated in the primary divergence among conspecific populations (e.g., Sunday and Hart 2013; Hart et al. 2014) and in the maintenance of reproductive boundaries between already diverged, hybridizing species in sympatry (e.g., Maroja et al. 2009). We describe the gene coding for bindin for all three sea star species in the genus *Pisaster* and analyze its molecular evolution within and between species. *Pisaster* bindin is similar in structure to that of other sea star genera studied to date and is highly variable in a central repetitive domain between the preprobindin sequence and the conserved functional core. We discover that evolution of the central repetitive domain among species may be partly influenced by the processes of concerted evolution within species, leading to differences in the copy number of repetitive domains among all three *Pisaster* species, homogenization of variation among paralogous copies within species, and divergence among haplotypes between species. Despite the potential effects of concerted evolution on within-species polymorphism, we find a signal of positive selection on bindin divergence in the second bindin exon among *P. ochraceus* alleles compared to *P. brevispinus*, with no significant genetic differentiation among alleles and current biogeographic patterns of selection and bindin variation that cannot easily be explained by ongoing reinforcement. Comparisons of codon model results among *P. ochraceus* populations consistently identified one site (codon 624) under positive selection and indicated diversifying selection among populations and across two terminal bindin lineages leading to alleles sampled in several populations.

The evidence for positive selection on conspecific bindin divergence within *Pisaster* species is limited to fewer lineages of alleles and fewer sites in the coding sequence in comparison with bindin population divergence in *Patiria miniata* (Sunday and Hart 2013; Hart et al. 2014). Several evolutionary hypotheses might account for the absence of a stronger signal of bindin divergence under positive selection at the level of conspecific populations: the absence of phenotypic selection, a relatively weak response to selection in the context of small population sizes, the homogenizing effects of gene flow on the signal of substitutions and selection acting on bindin, protein diversification by an evolutionary mechanism other than an excess of nonsynonymous differences among alleles that can be detected by codon models, or insufficient time for the effects of selection to manifest as bindin diversification. We find evidence supporting the latter two potential explanations for the relatively modest signal divergence under selection in *P. ochraceus,* and we consider how these findings may lend a more complete understanding of the source of bindin divergence in *Pisaster* species.

We found evidence of positive selection leading to bindin divergence among *P. ochraceus* populations allopatric and sympatric with *P. brevispinus*. The inference of positive selection on alleles sampled from allopatric populations, along with a lack of bindin differentiation among sympatric and allopatric populations of *P. ochraceus*, suggests that reinforcement during secondary contact may not be an important selective mechanism for bindin divergence among congeners. Patterns of positive selection that are inconsistent with reinforcement have also been shown in gamete recognition proteins of other species of marine invertebrates. Clark et al. (2007) found a signature of positive selection in two paralogs of the sperm acrosomal protein lysin in an abalone subspecies (*Haliotis tuberculata coccinea*) that does not coexist with any other congeners. Geographic variation of the sperm protein M7 lysin among three hybridizing *Mytilus* species also showed no obvious patterns of differentiation consistent with reinforcement as a major source of selection on this gene (Riginos et al. 2006). Among sea urchins, positive selection in bindin was detected among allopatric and sympatric populations of *Echinometra lucunter* and its sister species *Echinometra viridis,* across their geographic distribution (Geyer and Lessios 2009), and the allopatric congeners *Paracentrotus lividus* and *Paracentrotus gaimardi* show highly variable bindin sequences that are influenced by positive selection (Calderón et al. 2009, 2010). Similarly, phylogeographic analyses among both allopatric (Metz et al. 1998) and sympatric hybridizing species (Lessios et al. 2012) in the genus *Arbacia* identified no evidence of selection on bindin, suggesting a pattern that is also inconsistent with the hypothesis of ongoing reinforcement against hybridization.

Conservative population-level comparisons of positive selection could not single out any sites or branches with statistical support for *ω* > 1 among bindin lineages within *P. brevispinus*. These results suggest a lack of ongoing positive selection among bindin alleles within that species. In comparison with statistically significant divergence under selection among some *P. ochraceus* alleles, the absence of diversifying selection among *P. brevispinus* alleles is consistent with the predicted effect of lower spatial density of spawning adults and sperm-limiting conditions for fertilization, which are expected to decrease the potential for a sexual conflict over polyspermy (Levitan 1993, 2006).

Although we found statistically significant support for positive selection on bindin divergence among *P. ochraceus* populations, the small number of alleles and codons under selection is unexpected because *P. ochraceus* are characteristically found along shallow rocky shores in high-density aggregations during the summer months when spawning takes place, and this species seems more likely to experience high sperm concentrations, strong sperm competition, and strong selection by sexual conflict over fertilization rates (and polyspermy) similar to other intertidal species (e.g., abalone, Stephano 1992; Babcock and Keesing 1999). One potential explanation for the relatively small number of bindin codons under positive selection in *Pisaster* is the effect of concerted evolution on bindin divergence among conspecific alleles. Concerted evolution has been shown for other surface recognition molecules that interact with other proteins, including the egg receptor for abalone sperm lysin, pollen coat genes, fungal glycoproteins involved in immunorecognition, and egg extracellular coat proteins in *Drosophila* (e.g., Galindo et al. 2002; Fiebig et al. 2004; Johannesson et al. 2005; Carmon et al. 2007). The process of concerted evolution may lead to interspecific diversity in the copy number of repetitive units among species and ultimately leads to the elimination of substitution differences and homogenization of repeat structure within species through the molecular mechanisms of gene conversion or unequal crossing over at meiosis (Smith 1976; Dover 1982; Elder and Turner 1995; Swanson and Vacquier 1998; McAllister and Werren 1999; Carmon et al. 2010). We found evidence suggesting repetitive domain evolution by concerted evolution within species, as indicated by higher sequence similarity among *B* repeat domains within species than between repeat domains corresponding to the same region of the coding sequence among species. A second prediction of this hypothesis is that nucleotide substitutions in the terminal repeat domains should be maintained across populations of alleles and may lead to higher sequence divergence at the terminal repeats relative to other internal repeat domains (McAllister and Werren 1999; Carmon et al. 2007). The diversity in repeat structure in *P. ochraceus* was consistent with this prediction, with three internal *B* repeats (2*B*, 3*B*, and 4*B*) grouped into a single clade (Fig. 3b) and the highest nucleotide diversities observed across the C- and N-terminal repeat domains (Fig. 4). The lack of homogenization in repeat 1*B* in *P. brevispinus* is also consistent with concerted evolution within species, suggesting that repeat 1*B* has not been homogenized as *P. brevispinus* last shared a common ancestor with *P. ochraceus*. However, the highest bindin nucleotide diversity among all *P. brevispinus* populations sampled in this study was found only in the C-terminal region (Fig. 4).

While unequal crossover can be a major source of allelic diversity through reshuffling of existing variation or insertion and deletion of repeat copies within alleles (McAllister and Werren 1999; Meeds et al. 2001), gene conversion and the elimination of sequence polymorphism (within and between alleles) by concerted evolution effectively reduce the potential to detect the signal of diversifying selection within a population and across the entire length of a gene (Gay et al. 2007). Importantly, the observation that the site (codon 624) inferred to be under selection in *P. ochraceus* is positioned in the last repetitive domain and that this site is followed by a polymorphic indel of eight amino acids flanking the nonrepeating sequence at the C-terminal end of the second exon also supports the prediction that repeat copies at the end of a repetitive array should be less likely to experience gene conversion or homogenization by concerted evolution. Therefore, a signal of positive selection (*ω* > 1) averaged across all sites in a specific lineage is expected to be weak if an excess of substitution differences among alleles is likely to be limited to sites at the end of a repeating array. Interestingly, codon 624 in *P. ochraceus* occurs in the same part of the bindin structure as the positively selected codon 842 in two geographically isolated populations of the sea star *Patiria miniata* (Sunday and Hart 2013). Positive selection has also acted on polymorphism in the same corresponding gene region in sea urchin bindin, ∼40 codons upstream of the central core domain in six species from the genus *Echinometra* (Metz and Palumbi 1996; McCartney and Lessios 2002, 2004; Zigler et al. 2005; Vacquier and Swanson 2011) and among *Stongylocentrotus* species (Biermann 1998).

Positive selection for bindin divergence on the C-terminal end of the repetitive domain in *P. ochraceus* may underlie functional variation and sequence divergence in a potentially species-specific manner that is similar to the mechanism by which the vitelline envelope receptor for lysin (VERL) evolves in abalone species (Swanson and Vacquier 1998). VERL is a major egg coat glycoprotein and consists of 22 tandem repeats that are ∼153 residues in length (Swanson and Vacquier 1998; Galindo et al. 2002). While repeats 3–22 evolve neutrally and have been homogenized by concerted evolution, such that repeats within species are more similar to each other than to repeats between species (Galindo et al. 2002), sequence divergence and positive selection have been observed in the N-terminal repeats 1 and 2, suggesting that they evolve independently, and likely function in species specificity of sperm–egg interactions (Galindo et al. 2003; Clark et al. 2009).

In addition to the effect of the molecular mechanisms by which bindin evolves, historical population demographic effects can also influence the spatial distribution of adaptive variation in bindin and other coding sequences that might otherwise be expected to show a strong signal of divergence under selection. Tests of population divergence showed no significant differentiation in bindin among populations, even over large spatial scales, with no differences in the pattern of differentiation among *P. ochraceus* populations that are or are not sympatric with a congener. Similar patterns have been previously documented for COI spatial variation in *P. ochraceus* (Marko et al. 2010; Harley et al. 2006; Stickle et al. 1992; also see Pankey and Wares 2009) across the entire geographic range from California to Alaska, which includes two known biogeographic breaks found in other marine species from the northeastern Pacific (Dawson 2001; Sotka et al. 2004; Harley et al. 2006; Kelly and Palumbi 2010). Previous studies of population history using some of the same data (COI; Harley et al. 2006; Marko et al. 2010) consistently indicate that *P. ochraceus* has experienced significant population expansions over the last 20,000 years through recent recolonization from southern populations following the last glacial maximum. Although widespread allele sharing at phylogeographic loci has been largely interpreted as evidence of high levels of gene flow among populations by ongoing long-distance dispersal of larvae (Strathmann 1987; Harley et al. 2006), these findings suggest that relatively shallow demographic histories among northern *P. ochraceus* populations and incomplete lineage sorting of ancestral polymorphisms across the genome (see Marko and Hart 2011) may in part account for the genetic homogeneity in bindin sequences that we observed among populations. Our coalescent demographic analyses among adjacent *P. ochraceus* population pairs (that were not genetically differentiated) were congruent with this prediction and revealed surprisingly low gene flow parameter estimates among all populations (Table 6). Such estimates are also consistent with low rates of gene flow for bindin evolving under positive selection (relative to other groups of noncoding and nonreproductive loci) in the sea star *Patiria miniata*, which have been interpreted as population-specific selection against immigrant bindin alleles (*m*∼1 × 10^−6^; Hart et al. 2014). Taken together, these findings suggest that a combination of recent divergence, relatively large population sizes, and very low gene flow can explain the lack of bindin differentiation observed among *P. ochraceus* populations in a demographic context of relatively recent northern expansions along the northeastern Pacific coast.

A much stronger pattern of population-specific positive selection for bindin divergence has been identified in *Patiria miniata,* among two populations that occur north and south of a relatively old (∼280,000 years) phylogeographic separation in the northeastern Pacific (Keever et al. 2009; McGovern et al. 2010; Sunday and Hart 2013). Sunday and Hart (2013) found high allelic diversity and divergent selection for alternative bindin lineages and codons among populations in the face of low ongoing gene flow estimated from noncoding phylogeographic markers (McGovern et al. 2010). Because *P. miniata* does not coexist with any closely related species, positive selection for divergent bindin variation does not reflect reinforcement against hybridization. If divergence between *P. miniata* populations instead reflects selection based on sexual conflict within populations, then the contrast between this species and *P. ochraceus* may suggest that extended periods of time may be necessary for independent coevolutionary arm races among male and female gametes driven by sexual selection to produce patterns of high allelic diversity and bindin divergence between population that are detectable using codon models. This interpretation is directly relevant to our comparison between *Pisaster* species: High allelic diversity within all *P. ochraceus* populations sampled in this study (compared to generally low diversity at the mtDNA locus) is consistent with an arm races outcome of sexual conflict favoring selection for bindin polymorphism within populations (Gavrilets 2000; Gavrilets and Waxman 2002; Haygood 2004), but <20,000 years since the time of population splitting estimated for *P. ochraceus* in previous studies (e.g., Marko et al. 2010) may not be enough time for the effects of positive selection through sexual conflict to lead to population differences in bindin, especially for large-bodied sea stars with long generation times and long life spans of several decades (Menge 1975). Therefore, our analyses of bindin variation within and between species of *Pisaster* sea stars may indicate lower and upper bounds on the temporal scale on which the effects of selection on bindin may be most likely to be detected: >20,000 years (among *P. ochraceus* populations that show positive selection but no population divergence) and possibly not >280,000 years (between *P. miniata* populations that show strong positive selection and highly significant population bindin differentiation).

While this study aimed to distinguish between the effects of selection on bindin molecular evolution, functional studies identifying the correlation between intraspecific repeat variation (e.g., Minor et al. 1991) or specific amino acid differences (e.g., Levitan and Stapper 2009), and sperm-egg compatibility during fertilization are required for a more complete understanding of the role of concerted evolution and the selective mechanisms that mediate species-specific fertilization and bindin divergence within and between *Pisaster* species. Repetitive structure variation caused by concerted evolution and tandem duplication or deletion of complete repeat domains (and other smaller indel differences) in the mature bindin of *Pisaster* species suggests that these sea stars may experience selection on bindin divergence in a manner similar to bindin in other broadcast spawning marine invertebrates (e.g., Biermann 1998; Zigler and Lessios 2003; analogous, Moy et al. 2008; Sunday and Hart 2013). Similar variation in the number and length of repetitive domains flanking the core region and as few as 8–10 amino acid replacements have been shown to be associated with gamete incompatibility among congeneric species of sea urchins (Zigler et al. 2005). Experimental and molecular studies of pairs of coevolving sperm and egg recognition proteins are needed to effectively characterize the molecular interactions between male and female proteins (e.g., Clark et al. 2009; Tomaiuolo and Levitan 2010) and to sharpen our general understanding of how genetic changes are linked to ongoing coevolution and population-level divergence in sperm and egg compatibility.

### Data Archive

Full-length bindin sequences for all three *Pisaster* species (accession numbers: *P. ochraceus* KJ481933; *P. giganteus* KJ481934; *P. brevispinus* KJ481935) and population sets for *P. ochraceus* (accession number: KJ404124–KJ404193) and *P. brevispinus* (accession number: KJ404084–KJ404123) have been archived in GenBank.

## Supporting Information

**Appendix S1.** Posterior probability distribution plots for migration parameters; population genetic summary statistic including Tajima's *D*, population pairwise differences and AMOVA.

**Data S1.** Sixteen output files (M- and L-mode) from isolation-with-migration analyses in IMa.

**Data S2.** Eight input files for isolation-with-migration analyses in IMa.
