# Supplementary material for: Selection and demographic history shape the molecular evolution of the gamete compatibility protein bindin in Pisaster sea stars
Source: Ecol Evol. 2014 Mar 31;4(9):1567–88. doi: 10.1002/ece3.1042 (PMC4063459; doi:10.1002/ece3.1042)
Supplement: Supplementary file 1 [file ece30004-1567-SD1.pdf]

Appendix S1:

Figure 1. Posterior probability distributions of bidirectional migration parameter estimates between *P. ochraceus* population pairs

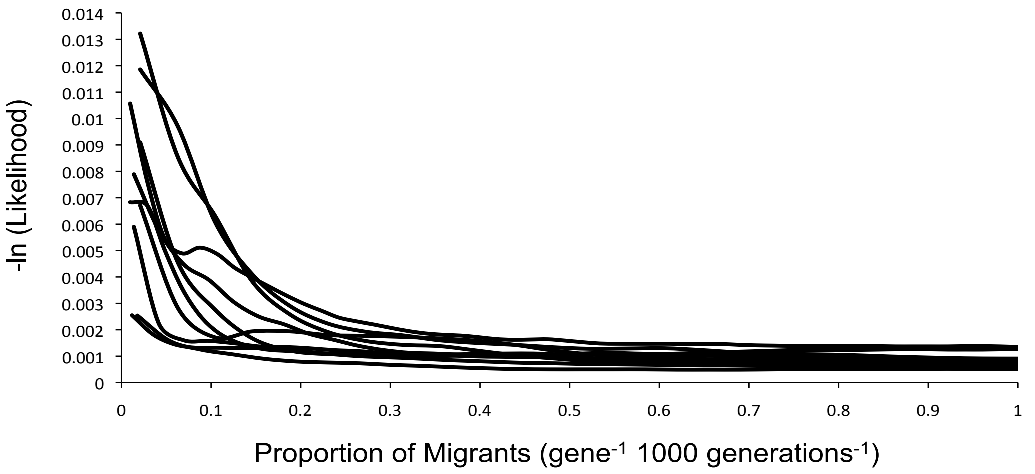

Table 1. Summary of samples obtained from the California Academy of Sciences, CA

Species	Accession Number	Location Sampled
P. brevispinus	149761	San Francisco Bay (SF Marina), CA
	157354A	San Francisco Bay (Alcatraz Island), CA
	157354B	San Francisco Bay (Alcatraz Island), CA
	162445	San Francisco Bay (Horseshoe Bay), CA
	162449	San Francisco Bay (Alcatraz Island), CA
	172146	San Francisco Bay (SF Marina), CA
P. giganteus	116600	Pillar Point (San Mateo County), CA

Table 2. Tajima's D statistics

Species	Population name	Tajima's D
P. ochraceus	Cordova, AK	-1.15169 NS
	Bamfield, BC	-1.06619 NS
	Lighthouse Park, BC	-1.05440 NS
	Hopkins, CA	-0.78153 NS
	La Jolla, CA	-0.90676 p=0.012
P. brevispinus	Bamfield, BC	0.54442 NS
	Port Moody, BC	-1.13785 NS
	SF Bay, CA	-1.63982 p=0.03

Table 3. Summary statistics for population pairwise differences (Φ_{ST}) and p-values:

a) ***Bindin***

P. ochraceus population pairwise Φ_{ST}

	COR	BAM	LHP	HOP
BAM	-0.02435			
LHP	0.00084	-0.04472		
HOP	0.00459	-0.05023	-0.02730	
LA	-0.02088	0.02088	0.04730	0.04830

Φ_{ST} p-values

	COR	BAM	LHP	HOP
BAM	0.71102			
LHP	0.38590	0.87467		
HOP	0.32472	0.97178	0.68439	
LA	0.68973	0.21632	0.09148	0.10306

P. brevispinus population pairwise Φ_{ST}

	BAM	PM
PM	0.05766	
CAS	0.07935	-0.00600

Φ_{ST} p-values

	BAM	PM
PM	0.10761	
CAS	0.09900	0.43966

b) ***COI***

P. ochraceus population pairwise Φ_{ST}

	COR	BAM	LHP	HOP
BAM	0.28179			
LHP	0.31160	-0.06937		
HOP	0.27785	0.02405	0.23612	
LA	0.26369	-0.11117	0.02773	-0.03292

Φ_{ST} p-values

	COR	BAM	LHP	HOP
BAM	0.07475			
LHP	0.05128	0.72775		
HOP	0.04445	0.39689	0.11553	
LA	0.14375	0.99990	0.44283	0.59895

P. brevispinus population pairwise Φ_{ST}

	BAM	PM
PM	0.01158	
CAS	0.09558	0.08527

Φ_{ST} p-values

	BAM	PM
PM	0.41372	
CAS	0.10692	0.23621
